# Enhancement of Withstand Voltage in Silicon Strain Gauges Using a Thin Alkali-Free Glass

**DOI:** 10.3390/s20113024

**Published:** 2020-05-26

**Authors:** Joon Hyub Kim, Ji-Hoon Han, Chan Won Park, Nam Ki Min

**Affiliations:** 1 Department of Nanomechatronics Engineering, Pusan National University, Busandaehak-ro 63 beon-gil 2, Geumjeong-gu, Busan 46241, Korea; kim4539@pusan.ac.kr; 2 Department of Control and Instrumentation Engineering, Korea University, Sejong-ro, Jochiwon-eup, Sejong-si 30019, Korea; dafory@korea.ac.kr; 3 Department of Electrical and Electronics Engineering, Kangwon National University, Gangwondaehak-gil, Chuncheon-si 24341, Korea

**Keywords:** strain gauge, silicon, alkali-free glass, high withstand voltage, micro-electromechanical system (MEMS), piezoresistive sensor

## Abstract

We present a cost-effective approach to produce silicon strain gauges that can withstand very high voltage without using any complex package design and without sacrificing any sensor performance. This is achieved by a special silicon strain gauge structure created on an alkali-free glass substrate that has a high breakdown voltage. A half-bridge silicon strain gauge is designed, fabricated, and then tested to measure its output characteristics. The device has a glass layer that is only 25–55 µm thick; it shows it is able to withstand a voltage of over 2000 V while maintaining a high degree of linearity with correlation coefficients higher than 0.9990 and an average sensitivity of 104.13. Due to their unique electrical properties, silicon strain gauges-on-glass chips hold much promise for use in advanced force and pressure sensors.

## 1. Introduction

Since the discovery of the piezoresistive effect in silicon (Si) and germanium in 1954 [1], there were continuous and challenging obstacles to overcome in the field of measuring basic mechanical quantities, such as stress, pressure, touch, acceleration, and weight. Si strain gauges were used to meet these challenges, and they are the main driving force behind the great progress we saw in piezoresistive sensing technology through the years [2,3,4]. In particular, Si micromachining techniques (including anodic bonding) played a major role in improving the performance of Si based piezoresistive sensors at large. The vast majority of commercial force and pressure sensors today use Si piezoresistive strain gauges. Typically, these are made from p-type Si and are either manufactured as separate elements for bonding to the surface of a sensing diaphragm [5,6,7,8,9] or embedded into an Si sensing membrane [10,11,12,13] using the Si micro-electromechanical system (MEMS) process.

Recently, high-performance Si gauge-based pressure sensors with a large sensing range, high sensitivity, and excellent insulating properties are in great demand due to their myriad potential applications ranging from industrial control systems to medical equipment. For example, industrial system air conditioners require pressure sensors that can withstand at least a test voltage of 1.8 kV for 1 s and 1 kV for 30 s at pressures higher than 30 bar [14]. Another example incudes medical pressure sensors in oxygen concentrator devices that need to have both a high degree of accuracy and a high degree of repeatability; on top of this, they should be able to measure oxygen tank pressure levels of about 2000 psi (136 bar) [15].

Currently, Si strain gauges for high-pressure applications are supplied without backing. Most of the unbacked gauges are not insulated on the bottom side, which makes them unsuitable for many industrial applications. Thus, existing Si gauges do not fulfil many of these new requirements at the chip level. In fact, some of these requirements can only be realized at package level by placing the sensing element inside a capsule filled with a silicone oil as a pressure transmission medium [16] or by embedding Si gauges in a glass frit within the recess formed in a metal diaphragm [17]. All previous approaches used a complex packaging structure that leads to high costs.

In this paper, we take an entirely new approach to completely meet all high-performance requirements at the chip level instead of creating expensive, complex workarounds at the packaging phase. This is accomplished by anodically bonding an Si wafer to an alkali-free glass wafer with a coefficient of thermal expansion (CTE) that is closely matched to that of Si wafer and that has a high breakdown voltage. We use deep reactive ion etching (DRIE) to form these Si strain gauges. The finished Si gauge chip is glass-frit bonded onto a metal diaphragm. This results in a product with reliable glass–glass frit bonding that, unlike the Si–glass frit bonding in existing technology, provides high bond strength and quality, while the CTE-matched Si–glass system can withstand very high voltages and has good electrical characteristics. The combination of these factors results in extremely high-performance Si strain gauges that are significantly better than any competitor. Furthermore, this gauge chip is easy to handle and use because of the thin glass backing. In a previous work [18], we reported preliminary results on Si strain gauges for high-withstand pressure sensors. At the time, the strain gauge sensor after glass frit bonding suffered from unpredictable reliability and quality, resulting in erroneous readings. We continued to revise fabrication processes and aging conditions to establish repeatability and reliability for high-quality strain gauges. It was found that the thermal and pressure aging after glass frit bonding has significant effects on the performance characteristics and reliability of Si gauges. We now present findings from the newly fabricated Si strain gauges.

## 2. Materials and Strain Gauge Design

**Figure 1:**
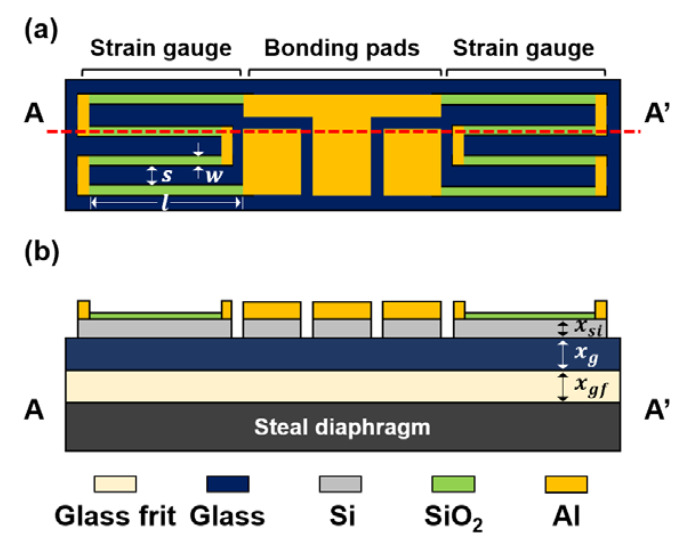
Layout (**a**) and cross-sectional view (**b**) of an Si strain gauge chip. The main design parameters are also illustrated: the length (l=210 μm), width (w=30 μm), distance between piezoresistors (s=100 μm), and shape of the piezoresistor along with the thicknesses of the gauge ($x_{si}=5\;\mu m$), glass ($x_{g}=25\;\mu m\;\mathrm{to}\;55\;\mu m$) and glass frit ($x_{gf}=30\;\mu m$).

Figure 1 shows a sketch of the half-bridge Si strain gauge chip featured in this work; it consists of two Si gauges and three Al bonding pads positioned on the glass substrate. Each gauge has four longitudinal piezoresistors where two resistors are connected by a short Al bar to minimize sensitivity degradation due to the effect of transverse stress. In order to obtain the highest sensitivity from the (100) Si layer, p-type piezoresistors can be easily arranged along the (110) direction. A piezoresistor 30 μm wide (w) and 210 μm long (l) was used to achieve the desired resistance and sensitivity. The distance between the piezoresistors was 100 μm (s).

**Figure 2:**
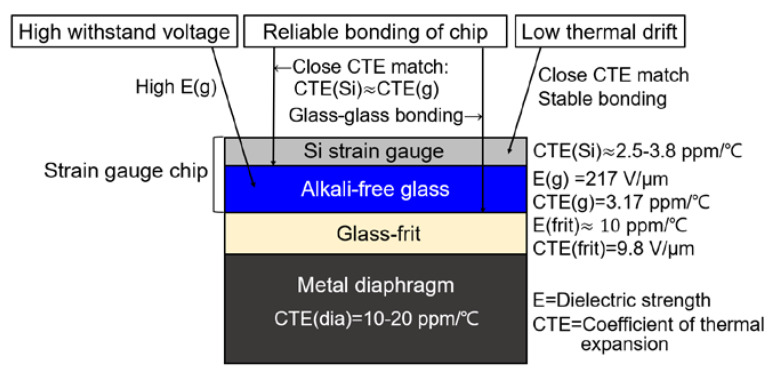
Summary of three main features for Si strain gauge design.

We focus on three main features in the following strain gauge design, as summarized in Figure 2. The first is to achieve a reliable withstand voltage beyond 2000 V. Frit glasses are special glasses with a particularly low softening point (below 550 °C). They have a low dielectric breakdown strength of approximately 9.8 V/μm compared to high-quality glass [19]. As such, we cannot realize a high breakdown voltage with only glass frit at the gauge level; instead, we need additional insulation for preventing leakage currents between the gauges and the metal diaphragm. The second feature is reliable bonding of the gauge to the metal diaphragm. Si has a specific thermal expansion in the range of 2.5–3.8 ppm/°C at the operating temperatures we are interested in; on the other hand, stainless steel and glass frit have a specific thermal expansion of around 10–20 ppm/°C and 10 ppm/°C, respectively. Due to the large CTE mismatch between the Si gauges and the kind of metal diaphragm to which the gauges are usually bonded, significant thermal stress can be generated within the gauges after the assembly cools down to room temperature. These residual thermal stresses generated during gauge bonding may have a significant impact on both the operation and performance of a gauge sensor. The introduction of thermal residual stresses often causes two serious problems: Si chip cracks and debonding from the metal diaphragm.

The third and final feature is the low output deviation and minimized temperature drift. As mentioned above, there is a large CTE mismatch between the Si gauges and the metal diaphragm. It can be clearly seen that this material mix inevitably leads to strain within the sensor if it goes through a temperature change. This strain will cause an offset change, and, when a high CTE material like glass frit is involved, this leads to temperature-dependent drift and hysteresis.

These three features can be realized by fabricating our Si strain gauge on glass substrate if an appropriate glass plate can be found for use as the substrate. Motivated by the discussion above, we chose an alkali-free soda-lime glass wafer. There are three reasons why we believe this is a good choice. Firstly, the glass substrate has a breakdown voltage 2.6 times higher than borosilicate glass. The dielectric strength of glass and glass frit is 217 V/μm and 9.8 V/μm, respectively [19,20]. This implies that the glass can be much thinner than regular glass frit while maintaining the same withstand voltage. Therefore, only a 25-um-thick glass substrate is needed to achieve a 2000-V-withstand-voltage Si strain gauge. Secondly, the coefficient of thermal expansion of this glass substrate (3.17 ppm/°C) is very much closer to that of Si material compared to glass frit that has a much higher CTE. Therefore, the thermal stress in the glass–glass frit bonded interface is much less than that in an Si–glass frit interface. Furthermore, glass–glass frit bonding is much stronger and more stable than Si–glass frit bonding due to compressive surface strengthening [21,22,23].

Finally, the existing Si gauge–glass frit–metal diaphragm systems used have a significant effect on the electrical characteristics of the gauge; one example is the temperature drift of Si strain gauges because of CTE mismatch between the Si, glass frit, and metal diaphragm. Variations in pressure and environmental temperature are transferred to the thin Si gauge through the high-CTE glass frit. On the other hand, in an Si gauge–glass plate–glass frit–metal diaphragm system, the thickness of the high-CTE glass frit is minimized, and the close CTE match between the Si and glass substrate makes the interface much less temperature-dependent than is the case for traditional Si–glass frit bonding. Many of the problems that normally occur when glass frit is used in the Si gauge–metal diaphragm bonding technique are eliminated by the use of our thin glass substrate between the Si gauge and glass frit.

## 3. Si Strain Gauge Fabrication

**Figure 3:**
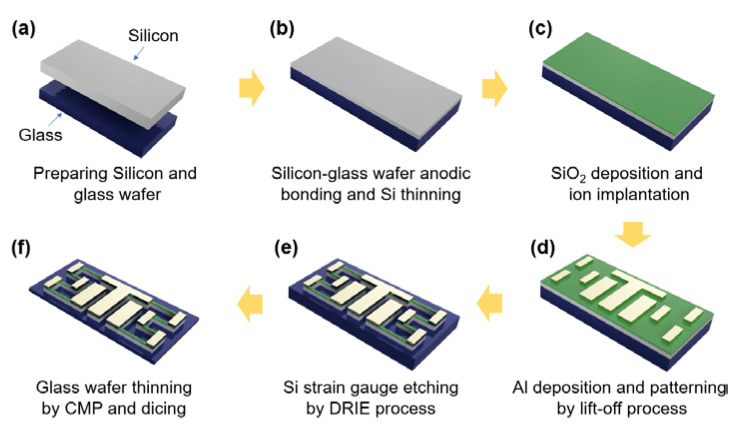
Fabrication process for Si strain gauge chip.

Figure 3 shows the main steps of the whole fabrication process for the Si strain gauge that has a high withstand voltage using the Si MEMS process. Due to temperature constraints on the glass, all processes were performed at temperatures below 600 °C.

The process started with an eight-inch Si wafer and an alkaline-free glass wafer with a high breakdown electric field. As the glass wafer is anodically bonded to the Si wafer, its CTE must be closely matched to that of the Si. Si–glass wafer anodic bonding (Figure 3b) was performed at a very high voltage of 1750 V at 500 °C since boro-aluminosilicate glass is alkaline-free [24]. The bond quality for anodically bonded wafers is critical because the bonded interface must be strong enough to withstand the following process steps, such as the mechanical thinning or dicing process that bonded wafers undergo. After anodic bonding, the Si wafer was thinned down to 5–10 μm using a conventional chemical mechanical polishing (CMP) process.

Next, a 500-Å-thick screen oxide was deposited on the thinned Si layer using low-pressure chemical vapor deposition (PECVD) at 350 °C. Sequential ion implantation of fluorine (F) ions (for pre-amorphization of the Si layer) and boron (B) ions (for p-type piezoresistors) was carried out with accelerating energies of 80 and 50 keV, respectively. The doses of F and B ions were $3\times 10^{15}$ and $5\times 10^{14}$ ions/cm^2^, respectively. Post-implantation annealing for dopant activation was performed at 600 °C for 2 h, where an average sheet resistance of 193 Ω/sq was formed (Figure 3c). Al (800 nm)/Ti (100 nm) metallization was performed using the lift-off technique (Figure 3d). Individual layers were deposited by radio frequency magnetron sputtering without vacuum break. The thinned Si layer was patterned using deep reactive ion etching (DRIE) to form the Si strain gauges and the bond pads. This etch was performed over 90 s on a 5-μm-thick Si layer. An SiO_2_ (500 Å)/SiN (2000 Å) layer for gauge passivation was deposited using PECVD at 350 °C, and a passivation layer was etched on the metal pads by RIE to form wire bonding contact points (Figure 3e).

Finally, the backside glass was thinned down to about 25–55 µm by CMP, and then the whole wafer was diced into strain gauge chips using a sawing machine (Figure 3f). For comparing the breakdown voltage only, Boro 33 glass-based Si strain gauges were also fabricated using much the same process as in the steps described above.

**Figure 4:**
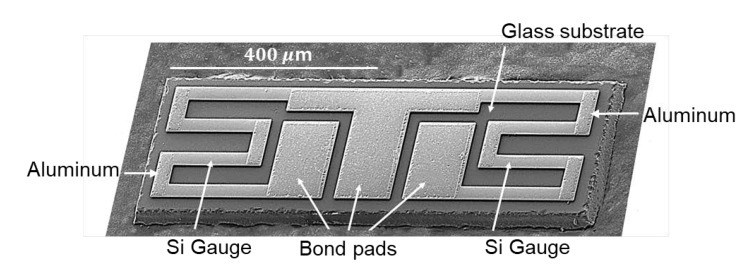
SEM micrograph of diced half-bridge chip with two Si strain gauge elements and three bond pads.

An SEM image of the manufactured Si gauge chip is shown in Figure 4. No cracks or defects were found on the diced chip. However, one edge of chip was quite rough due to the difficulty of dicing amorphous glass in comparison with Si; this edge in no way affects gauge operation. It can be seen that the Si gauge on both sides of the chip was well formed with straight lines, and the contact opening for wire bonding was developed in the center of the three bonding pads. The width, length, and initial resistance of the Si gauge were 31 μm, 726 μm, and 4.4 kΩ, respectively.

## 4. Results and Discussions

### 4.1. Glass Frit Bonding and Assembly

**Figure 5:**
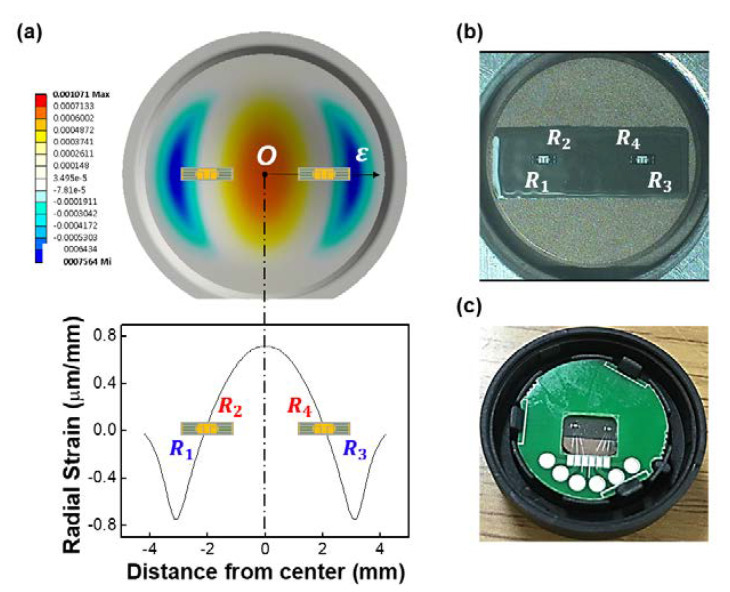
(**a**) Simulation result of the radial strain distribution in the metal diaphragm for each pressure loading condition by ANSYS software; (**b**) Si strain gauges bonded to metal diaphragm; (**c**) Al wire-bonded gauge chips assembled into the plastic package for measurements.

The traditional metal strain gauges for use on diaphragm pressure transducers have circular and linear grid elements for sensing the tangential and radial strain fields, respectively [25]. However, the Si gauges are designed as linear grids, as shown in Figure 1a and Figure 5a, to take advantage of the orientation of the radial strain (ε). The main advantages of using a linear gauge configuration are ease of fabrication and generally lower gauge cost. The radial stress distribution in the metal diaphragm for each pressure loading condition was obtained from ANSYS finite element method (FEM) software. As shown in Figure 5a, the radial strain (ε) decreases rapidly as the radius increases, becoming negative and approximately equal to the center strain at the edge. These values were used to calculate the average strain experienced by each strain gauge.

In order to evaluate the electrical performance of the Si strain gauge, two-gauge chips were bonded using glass frit [26,27] along the radial direction of a circular metal diaphragm, as shown in Figure 5b. After bonding, the sensing elements (Figure 5b) were subjected to the following temperature cycle: thermal aging → pressure aging → calibration. Firstly, thermal aging was carried out for 72 h at 160 °C, followed by 100-bar pressure aging for 30 min at 25 °C. During calibration testing, the data were taken at applied pressures of 10, 20, 30, 40, and 50 bar. The temperature profile was 25, 0, −40, 0, 25, 40, 80, 125, 80, 40, and 25 °C. Next, the sensing elements (Figure 5b) and printed circuit board (PCB) were assembled into the lower housing using black epoxy for complete fixation. Then, the interconnections between the gauge dies and the PCB was made using wedge-bonded aluminum wires (bonder: BONDJET-BJ820; Hesse & Knipps Inc.,CA, San Jose, USA), as shown in Figure 5c. Gauges 1 and 3 were subjected to compressive stress and gauges 2 and 3 were subjected to tensile stress when pressure was applied. Several experiments were conducted to evaluate the electrical performance of the Si strain gauge.

### 4.2. Dielectric Withstand Voltage

Withstand voltage is defined as the maximum voltage required to produce a dielectric breakdown of the device. A dielectric withstand voltage test verifies that the insulation of a component or device is sufficient to protect the operator from electrical shock. Here, the dielectric breakdown properties of Si strain gauges fabricated on alkali-free glass substrate (Eagle XG glass; Corning Inc., New York, NY, USA) were studied and contrasted with those of alkali-containing boro-aluminosilicate (Boro 33; Schott Inc., Mainz, Germany), which is commonly used in MEMS sensor packaging.

An alternating current (AC) withstand test was conducted using the GPI-825 Hipot tester (GW Instek Inc., New Taipei City, Taiwan). The Hipot tester connects one side of the supply to a safety ground (metal diaphragm), while the other side is connected to the strain gauge electrodes being tested. With the supply connected like this, the proposed strain gauges were firstly tested to endure up to 1.5 kVAC for 60 s; then, they were tested to breakdown using 100-V steps. This process was applied repeatedly and sequentially for all samples until breakdown occurred.

**Figure 6:**
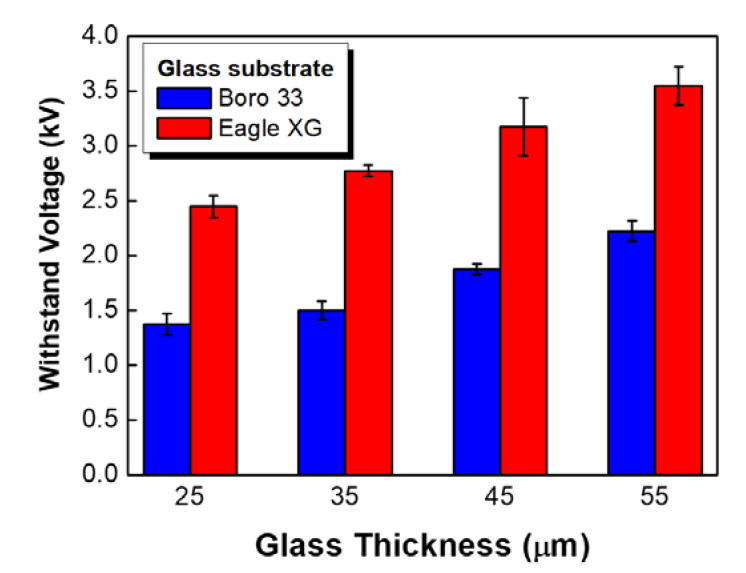
Withstand voltages of Si strain gauges with four different glass thicknesses; gauges were fabricated on two different glass substrates represented by red and blue (number of samples = 50).

Figure 6 shows the dependence of breakdown voltage on glass thickness at room temperature after bonding to a metal diaphragm. For example, the strain gauges on 25-μm-thick alkali-free glass substrate were found to withstand 1.5 kVAC for 60 s and to break down at 2.4 kVAC after 65 s, which fully meets the requirements for pressure sensors in the heating, ventilation, and air conditioning (HVAC) industry. On the other hand, for Si gauges with 25-μm-thick Boro 33 glass, electrical breakdown occurred at 600 V after 1 s.

**Figure 7:**
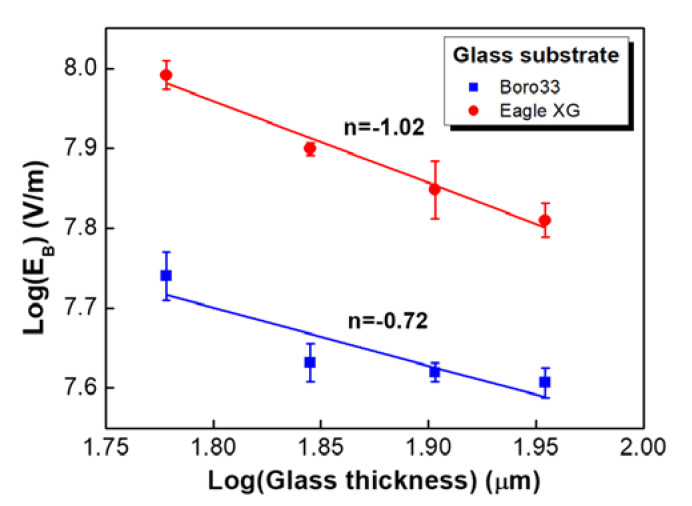
Breakdown strength of glass frit-bonded strain gauges as a function of glass thickness.

The maximum breakdown voltage of the material is more frequently expressed as dielectric field strength (volts per unit thickness), which is a measure of the electrical strength of a material as an insulator. A higher dielectric strength represents a better quality of insulator. Figure 7 shows the variation of the dielectric breakdown field of Si strain gauges bonded to a metal diaphragm as a function of device thickness; this relationship is well fit to the following well-known expression [28,29]:

(1) $$E_{B}\;\propto \;x^{-n},$$

where $E_{B}$ is the breakdown field strength, x is the glass thickness, and n is the fitting parameter. Although insulating materials are very different and the failure mechanisms may not be the same, it is well known that Equation (1) shows a general empirical relationship between the breakdown strength, $E_{B}\;$ and the thickness of the dielectric. This relationship fits data for amorphous solids, such as silica, glass, and polymers, although the values of *n* are not unique. As shown in Figure 7, the Si strain gauge on alkali-free glass exhibited a remarkably high characteristic breakdown strength, vastly exceeding that of the alkali glass. The Boro 33 glass with high sodium content had a much smaller breakdown field, supporting the supposition that, here, ions play a dominating role.

The n factor can be varied with experimental configuration, and it may be related to microscopic structure and charge transfer. In general, alkali-free glasses show two breakdown regimes corresponding to n ~ 0.1 in the lower thickness range and n ~ 1 for higher thicknesses; this implies two different breakdown mechanisms with a transition around 20-μm-thick glass [28,30]. In our case, all data are described well by Equation (1) for a total thickness between 25 and 55 μm, while the slope, n=−1.02, is very close to the prediction given for thermal breakdown. Furthermore, the breakdown field strength increases substantially with decreasing glass thickness, indicating heat breakdown (interrelated increase in temperature and electrical conductivity) as the preferred breakdown mechanism [28].

### 4.3. Sensitivity of Si Strain Gauge

The sensitivity S of the gauge to strain state can be calculated from the relative variation (ΔR/R) in the gauge resistance and the radial strain (ε) [31].

(2) $$S=\frac{\Delta R}{R}/\varepsilon .$$

**Figure 8:**
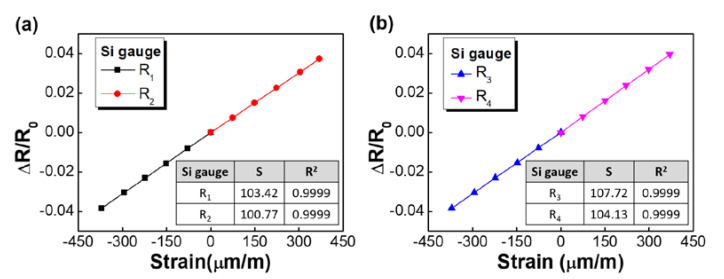
Strain sensitivities of the Si strain gauges fabricated on alkali-glass substrate. (**a**) $R_{1}$ and $R_{2}$, (**b**) $R_{3}$ and $R_{4}$. (The insets summarize the typical sensitivities and linear correlation coefficients)

Each strain gauge was tested over five runs in a temperature (−40 °C to 125 °C) and humidity (relative humidity of 40%) chamber capable of maintaining a heat–cool cycle within a specified range while providing electrical connection to the test device. The resistance changes of strain gauge were measured with digital multimeters and recorded simultaneously, in real time, by a computer as the devices were tested. The relative changes in resistance were calculated in order to find the gauge sensitivity. As shown in Figure 5a, gauges $R_{2}$ and $R_{4}$ experienced a tensile strain and an associated increase in resistance. On the other hand, $R_{1}\;$ and $R_{3}$ were compressed, which led to a resistance decrease. The measured (ΔR/R) values were plotted against the radial strain for the four resistors, as shown in Figure 8. In each plot, linear curve fitting was added. The Si gauges demonstrated a linear relationship with changes in resistance versus strain for a large range of radical strain. The data showed a high degree of linearity with correlation coefficients higher than 0.9990 for all the Si strain gauges. The sensitivity S was obtained from the slope of the curves using Equation (2) for the four strain gauges. For example, the average sensitivity of R_4_ was calculated to be 104.13 with a standard deviation of 0.06, whereas the standard uncertainty was found to be 0.012 over five test runs with 20 different gauges from the same batch.

### 4.4. Bridge Output Characteristics

Strain gauge type transducers such as load cells and pressure sensors usually employ a Wheatstone bridge configuration with one active gauge in each arm of the bridge to detect minute changes in resistance corresponding to the amount of strain they undergo. To convert these small changes of resistance experienced by the gauge in response to applied pressure into an output voltage, two half-bridge chips were configured with Wheatstone bridges.

**Figure 9:**
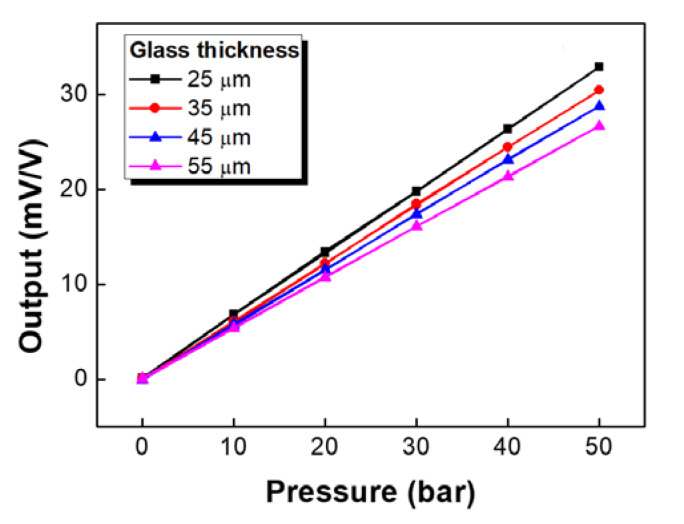
Wheatstone bridge output for Si strain gauges with various glass thicknesses.

Figure 9 shows the room temperature output characteristics of Wheatstone bridges composed of strain gauges with various glass thicknesses. The proposed pressure sensors exhibited linear output characteristics regardless of the thickness of the glass substrate. In addition, the span decreased relatively linearly with an increase of glass substrate thickness because the strain transmitted from the diaphragm to the Si gauge decreased as the glass substrate became thicker. Span deviations for samples with same thickness depend not only on the variation of the single element resistance with strain, but also on several factors such as misalignment of gauge chips, quality of glass frit bonding, non-uniformity of glass frit, and metal diaphragm thickness, as well as the measurement circuit [14].

**Figure 10:**
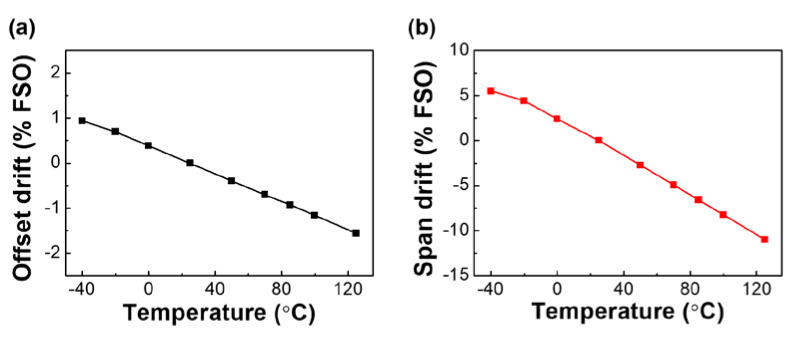
Offset (**a**) and span (**b**) drifts over temperature of bridge circuit outputs.

A major problem associated with a four-arm Wheatstone bridge circuit is the inherent cross sensitivity to temperature. The influence of temperature on an Si gauge sensor is exhibited by a change in the span and offset of the sensor output, as shown in Figure 10. It was found that the thermal and pressure aging after glass frit bonding has significant effects on the performance characteristics and reliability of Si gauges. The two gauges in Figure 10 have the same structure except that different conditions of thermal and mechanical aging are used. The newly fabricated Si strain gauges (used in this work) showed much more linear and predictable thermal drift than the previous gauges [18], indicating that these characteristics can be easily temperature-compensated by using commercial application-specific integrated circuit (ASIC). The offset drift is a straight line, which has a maximum of 1.5% FSO (full-scale output). The span drift decreases with a slope of −0.153% FSO/°C, except for a slight bow at low temperature, with increasing temperature.

**Figure 11:**
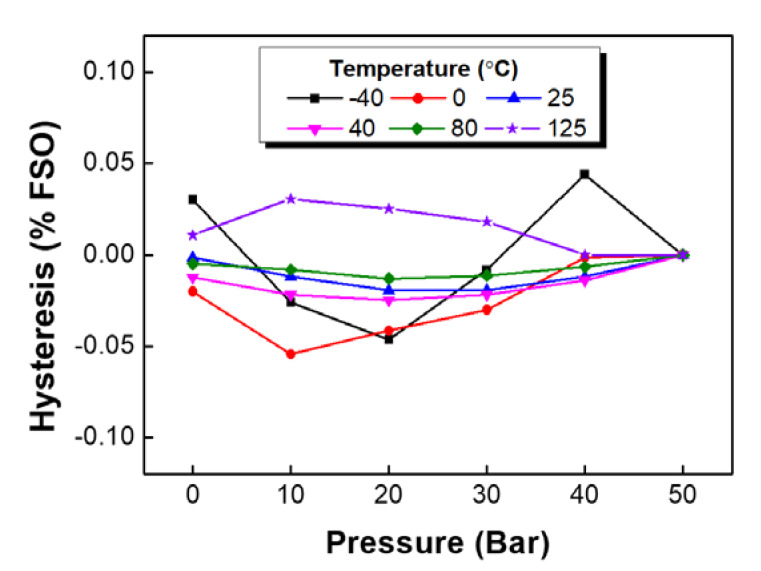
Hysteresis of a bridge circuit outputs at various temperatures and pressures.

Figure 11 shows temperature-dependent hysteresis at the four-arm gauge bridge. We did not find a correlation between the hysteresis and the temperature, although the hysteresis error was very low (less than ± 0.05).

### 4.5. Current Application of the High-Withstand-Voltage Si Strain Gauges

**Figure 12:**
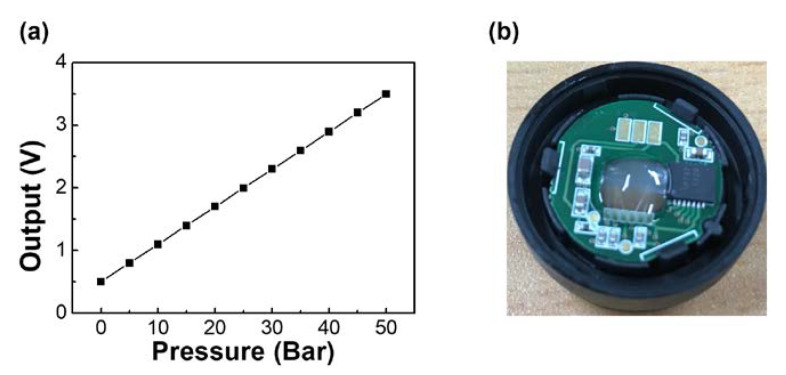
(**a**) Output characteristics of temperature-compensated pressure sensor measured at 25 °C under pressure ranging from 0 bar to 50 bar, (**b**) assembled the sensing element and customized ASIC into plastic housing.

Here, we demonstrate a practical application of our strain gauge elements presented in the previous section. As mentioned in the introduction, industrial system air conditioners need two high-pressure sensors (range: 0–30 bar and 0–50 bar) that can withstand at least a test voltage of 1.8 kV for 1 s and 1 kV for 30 s. Furthermore, the pressure sensor for air conditioning requires a much higher accuracy of ±0.5% FSO or less than that for automobiles (typically 1.5–2% FSO). The sensing element (Figure 5b) and a customized ASIC (ZSSC3138; ZMDI, Dresden, Germany) were assembled into the plastic housing, as shown in Figure 12b. The pressure sensor was calibrated and temperature-compensated using ASIC, which contains the temperature sensor in the chips. The sensor outputs were measured for pressure ranges up to 50 bar at 25 °C. Figure 12a shows a temperature-compensated output curve of prototype pressure sensors with high withstand voltage designed for system air conditioners, exhibiting almost no deviation from the ideal linear curve. The prototype pressure sensor performed well within a range of measured pressure, showing a 12 mV/V/bar sensitivity, a 0.30% FSO accuracy error, a 0.10% FSO linearity, a hysteresis less than 0.11% FSO, and a 0.25% FSO thermal drift.

## 5. Conclusions

We demonstrated an Si strain gauge with a very high withstand voltage. The gauge was fabricated on an Si-on-glass substrate using a combination of Si–glass wafer bonding and DRIE etching steps. The newly designed strain gauge sensors were found to break down after 2.4 kVAC was applied for 65 s, which fully meets the high-withstand-voltage requirements for diaphragm-type pressure sensors in industrial applications. They also show excellent output properties in terms of linearity, sensitivity, and accuracy. In addition, Si gauge-on-a-glass chips are easy to handle due to the presence of a glass substrate. Ongoing work is focused on optimizing the Si strain gauge design and glass frit bonding process for use in the manufacture of press sensors.
